# The impact of the COVID-19 pandemic on referrals to liaison psychiatry services at University Hospital Hairmyres, NHS Lanarkshire

**DOI:** 10.1192/bjo.2021.173

**Published:** 2021-06-18

**Authors:** Cristina Paton, James Hills, Rekha Hegde

**Affiliations:** University Hospital Hairmyres

## Abstract

**Aims:**

The COVID-19 pandemic has led to sweeping public health restrictions with predictable impact on mental health. In Scotland, lockdown measures during the first wave of the pandemic commenced on 23rd March 2020 and only began to ease after 29th May 2020. The aim of this study was to evaluate the impact of the first wave of the COVID-19 pandemic on the number and type of referrals made to the adult psychiatric liaison nursing service (PLNS) at University Hospital Hairmyres, NHS Lanarkshire.

**Method:**

We collated all of the archived referrals made by our local emergency department to the PLNS at University Hospital Hairmyres for adults (aged 18–65 years) during the period of the first COVID-19 national lockdown (April-July 2020) and the corresponding period one-year prior (April-July 2019) to analyse differences in referral numbers and demographics. Additionally, for referrals made during 2020, we conducted a qualitative review of electronic records to determine the reason for referral, contributory stressors to presentation, and in particular any effect from COVID-19.

**Result:**

A total of 549 referrals were made over the study period, with 320 in 2019 and 229 in 2020, a decrease of almost 30%. In 2019, referrals fell each month from April (n = 89) to July (n = 74), while this trend was reversed in 2020, rising from April (n = 45) to near-usual levels by July (n = 68). Compared to baseline, referrals in April 2020 were for a higher proportion of men (62.2%). On qualitative analysis, 26 records (11.3%) could not be found. Otherwise, the most common reasons for referral were suicidal ideation (43.3%) and/or deliberate self-harm (39.9%). Many patients presented with comorbid substance misuse (54.2%) and the majority were not known to community services (64.5%). COVID-19 was implicated in 48 referrals (23.6%), but only 2 of these arose as a direct result of infection.

**Conclusion:**

We have observed clear differences in the pattern of referrals made to the adult PLNS during the first COVID-19 national lockdown. COVID-19 was implicated in a minority of referrals, but most were related to secondary effects of lockdown restrictions rather than COVID-19 infection. Possible reasons for fewer referrals during this time could be non-presentation through fears of contracting COVID-19 or altruistic avoidance of putting “pressure on the NHS”. Further studies would be insightful; in particular, equivalent analysis of contacts with community services; and qualitative patient perspectives regarding reasons for non-presentation during this time.

